# Optimizing Nurse Rostering: A Case Study Using Integer Programming to Enhance Operational Efficiency and Care Quality

**DOI:** 10.3390/healthcare12242545

**Published:** 2024-12-17

**Authors:** Aristeidis Mystakidis, Christos Koukaras, Paraskevas Koukaras, Konstantinos Kaparis, Stavros G. Stavrinides, Christos Tjortjis

**Affiliations:** 1 School of Science and Technology, International Hellenic University, 14th km Thessaloniki-Moudania, 57001 Thessaloniki, Greece; a.mystakidis@ihu.edu.gr (A.M.); p.koukaras@ihu.edu.gr (P.K.); 2 Department of Physics, Democritus University of Thrace, University Campus, St. Lucas, 65404 Kavala, Greece; ckoukara@physics.duth.gr (C.K.); sstavrin@physics.duth.gr (S.G.S.); 3 School of Business Administration, University of Macedonia, 156 Egnatia Street, 54636 Thessaloniki, Greece; k.kaparis@uom.edu.gr

**Keywords:** nurse rostering, integer programming, healthcare management, operational efficiency, oncology departments, health informatics, healthcare analytics

## Abstract

**Background/Objectives:** This study addresses the complex challenge of Nurse Rostering (NR) in oncology departments, a critical component of healthcare management affecting operational efficiency and patient care quality. Given the intricate dynamics of healthcare settings, particularly in oncology clinics, where patient needs are acute and unpredictable, optimizing nurse schedules is paramount for enhancing care delivery and staff satisfaction. **Methods:** Employing advanced Integer Programming (IP) techniques, this research develops a comprehensive model to optimise NR. The methodology integrates a variety of constraints, including legal work hours, staff qualifications, and personal preferences, to generate equitable and efficient schedules. Through a case study approach, the model’s implementation is explored within a clinical setting, demonstrating its practical application and adaptability to real-world challenges. **Results:** The implementation of the IP model in a clinical setting revealed significant improvements in scheduling efficiency and staff satisfaction. The model successfully balanced workload distribution among nurses, accommodated individual preferences to a high degree, and ensured compliance with work-hour regulations, leading to optimised shift schedules that support both staff well-being and patient care standards. **Conclusions:** The findings underscore the effectiveness of IP in addressing the complexities of NR in oncology clinics. By facilitating a strategic allocation of nursing resources, the proposed model contributes to operational excellence in healthcare settings, underscoring the potential of Operations Research in enhancing healthcare delivery and management practices.

## 1. Introduction

Operations Research (OR) applies advanced analytical methods to optimise decision-making in complex organizational contexts. One such challenge is the Nurse Rostering Problem (NRP), where the allocation of nursing staff is vital for both patient care and operational efficiency. In oncology departments, efficient nurse scheduling is essential to ensure continuous, high-quality care [1,2,3,4].

To address this, recent OR research integrates mathematical models with healthcare management. Solutions often involve mathematical optimisation, including Integer Programming (IP), which offers a robust framework for improving NR in dynamic clinical settings. However, the use of such models in oncology clinics presents new obstacles and potential. Oncology departments have unpredictable demand patterns and a vital requirement for specialised treatment, which requires a flexible and strong rostering method [5]. Furthermore, the well-being of nursing staff, a vital component of sustainable healthcare delivery, must be included in the optimisation process, guaranteeing schedules that are not only efficient but also equitable and attentive to individual nurses’ needs [6].

The method used in this work is mathematical optimisation, focusing on accuracy and simplicity. In order to maximise operational efficiency, the optimal solution under the specified constraints is found, using mathematical formulations, such as linear and IP, whose integration attempts to distribute the work hours among nursing staff in a real case study.

The extension of the current model to incorporate Machine-Learning (ML) techniques offers an innovative approach to decision-making processes traditionally addressed through heuristics. For instance, employing supervised ranking for specific inputs during the subsidiary Mixed-Integer Programming (MIP) phases improves the overall performance of MIP solution mechanisms, aligning with studies indicating the feasibility of such applications [7,8]. Moreover, to improve NR efficiency and decision-making, the conceived approach can benefit from incorporating the modelling of complex information networks [9] to analyse the intricate interactions between nurses, patients, and various clinical factors. Such an approach can use bi-functional ML algorithms tailored to the dynamic nature of healthcare settings.

Finally, using linear regression to predict the annual number of shifts per nurse, coupled with data storage for weekly updates, facilitates adaptive learning by the program, while aiming to correct nurse scheduling over time to balance their workload throughout the year.

### 1.1. Problem Statement

Oncology departments’ NR is a classic operational challenge, driven by the requirement to match healthcare delivery to patient needs. Oncology patients require expert treatment and continuity, making this challenge even greater [3,10]. The traditional scheduling paradigms, often reliant on manual processes and heuristic decision-making, fall short in addressing the multifaceted dimensions of this problem, leading to inefficiencies that impact both patient care and staff welfare [4,11,12]. However, existing methodologies struggle to incorporate real-time data and adapt to sudden changes, resulting in either overstaffing, which strains resources, or understaffing, which compromises care quality and increases stress on the nursing staff [5].

The introduction of IP and other mathematical optimisation techniques offers a viable solution. These models can create flexible scheduling systems that better address the complexities of NR in oncology departments [7]. These advanced models have potential, but applying them in real-world cases is challenging due to the complications of effectively reflecting the nursing workforce’s limits and preferences and oncology patient care dynamics.

The contribution of this research lies in revealing how OR challenges are addressed in real healthcare settings. It demonstrates that theoretical models must be adapted and refined to effectively solve operational inefficiencies in healthcare institutions, thereby bridging the gap between theory and practice.

### 1.2. Integrating OR and Healthcare Management

This paper aims to critically examine the optimisation of rostering processes at a hospital in Greece, addressing the complexities of healthcare staffing. IP coupled with IBM’s Cplex program [13], via the Java Iloplex library [14], plays a pivotal role in navigating these challenges. The methodology utilises a dual-strategy approach: applying all constraints initially and subsequently relaxing some to explore alternative solutions. This iterative process highlights the flexibility and adaptability essential in developing efficient rostering systems, emphasizing the importance of stakeholder engagement.

The primary objectives of this work, employing OR in healthcare management, are summarised below:

1. Analyze Existing Rostering Practices: Investigate current NR practices to identify inefficiencies and areas for improvement, creating a foundation for the application of mathematical optimisation techniques to address identified gaps.
2. Integrate IP Models: Explore the use of advanced IP models in NR, aiming to build a system with enhanced flexibility, efficiency, and adaptability. These models will balance the operational demands of oncology care with the preferences and well-being of nursing staff.
3. Enhance Operational Efficiency: Quantify the impact of IP-based rostering systems on operational efficiency, providing evidence through the literature on the practical benefits of optimisation techniques.
4. Promote Staff Satisfaction: Adhering to labour regulations, workload balance, equitable rostering, and personal constraints, the proposed OR solution indirectly aims to create a supportive working environment for nursing staff.
5. Develop a Scalable and Adaptable solution: Create a flexible rostering solution adaptable to various oncology departments, providing a model for systemic improvements in healthcare rostering practices.

## 2. Background

The origins of OR can be traced back to 1665, when Newton’s method for solving differential equations was used. However, OR’s major advancements occurred in the 20th century, particularly during World War II, when British and American military forces formed interdisciplinary teams to address wartime operational challenges. This period marked the official recognition of OR and demonstrated the effectiveness of scientific methodologies in solving complex problems [15].

Early efforts to address NRP primarily involved heuristic methods and basic mathematical programming [2,16]. These methods, although foundational, were eventually replaced by more sophisticated optimisation techniques, particularly Mixed Integer Linear Programming (MILP). These approaches facilitated the balancing of strict staffing requirements with nurse preferences, optimizing shift allocations and improving operational efficiency. MILP has been particularly effective in addressing the variable demands of oncology departments, where patient loads can fluctuate unpredictably [17,18].

The development of potent optimisation libraries like CPLEX and Gurobi [19] has accelerated the evolution of OR from theoretical frameworks to computational problem-solving. These tools, integrated into programming environments like Java and Python, have significantly enhanced the ability to apply IP to complex problems such as the NRP. This technological shift has democratised access to advanced methodologies, enabling their application across various healthcare settings.

### 2.1. Definition and Challenges of Integer Programming

LP is a methodological cornerstone in OR that focuses on maximising or minimising a linear objective function under a set of linear constraints [2]. LP’s foundational role is pivotal in addressing a wide array of optimisation challenges across diverse fields, from resource allocation to financial planning, providing a structured approach to decision-making [18].

IP emerges as a specialised extension of LP, where decision variables are constrained to integer values, reflecting the discrete nature of many real-world problems. This distinction is crucial in scenarios where fractional solutions are infeasible or lack practical significance, such as scheduling, routing, and allocation tasks [20]. Pure Integer Programming (PIP) and MILP further expand the applicability of IP by accommodating purely integer and mixed (integer and continuous) variables, respectively, thus offering a more versatile toolkit for modelling complex systems [21,22].

Binary decision variables, a subset of IP, introduce the ability to model decisions in a binary format (e.g., yes/no, on/off), enabling the precise representation of choice-based constraints and logical conditions [23]. This binary structure is instrumental in formulating problems where decisions are inherently dichotomous, enhancing IP’s utility in designing efficient and effective solutions [18].

Despite IP’s rigid framework for addressing optimisation problems, the complexity of solving IP models increases exponentially with problem size, owing to the combinatorial explosion of possible solutions. This complexity necessitates the development of sophisticated solution techniques, such as the Branch and Bound and Branch and Cut algorithms, which systematically explore and prune the solution space to identify optimal or near-optimal solutions [18,24].

Cutting-plane methods are another example of how IP solution strategies are always changing. They provide a strong way to make IP models more specific by repeatedly adding constraints that get rid of parts of the solution space that can not be solved without leaving out any possible integer solutions [25].

### 2.2. Integer Programming Workforce Problems

The search for more effective scheduling techniques has been a result of the dynamic nature of workforce management, which is characterised by fluctuating employment levels and the need for flexible planning strategies. Innovations such as job rotation and part-time work have emerged as responses to these fluctuating demands, reflecting the evolving landscape of labour arrangements [3,4]. The seminal contributions by [2] introduced IP and MIP into workforce planning, marking a pivotal shift towards more sophisticated and flexible scheduling models.

The subsequent application of IP and MIP to address complex scheduling issues within organizations has been documented by [10,26,27]. These approaches have yielded mathematical models that optimise schedule management and resource allocation, enhancing the adaptability of workforce planning to changing conditions.

Further advancements in this domain, as demonstrated by [28,29,30], highlight the transformative impact of IP and MIP on improving job satisfaction, minimising employee stress, and boosting overall productivity and service quality. By refining shift scheduling and workload distribution strategies, these models contribute to creating a more equitable and satisfying work environment.

### 2.3. Mathematical Models of Workforce Planning

Mathematical modeling can address the challenges of workforce scheduling. In nurse scheduling, it can be modelled as a 0–1 shortest path network problem. This model was applied, effectively utilizing IP to optimise the allocation of nursing staff within healthcare settings, ensuring that operational demands were met efficiently and effectively [31].

Building on this foundation, hierarchical workforce scheduling was explored, devising mathematical models to delineate the organisation of staff based on skill levels and operational requirements [26]. These models facilitate the understanding of scheduling dynamics. Here, the flexibility for higher-skilled workers to substitute for lower-skilled ones is crucial, accommodating variable daily workloads and ensuring employees receive mandated days off. The objective function defined by [26] emphasises cost minimisation across the workforce composition (Equation (1)):

(1) $$\min\sum_{k=1}^{m}C_{k}W_{k}$$

where $W_{k}$ = Number of workers of type *k*, $C_{k}$ = Cost of type *k* worker.

Expanding upon Billionnet’s framework [26], another model was introduced accommodating diverse shift lengths and work patterns [28]. This adaptation allows for a more granular optimisation of workforce schedules, aligning shift assignments with operational demands and worker preferences. Their revised objective function, incorporating shift-specific costs, showcases the model’s flexibility in addressing the scheduling complexities (Equation (2)):

(2) $$\min\sum_{b=1}^{B}\sum_{k=1}^{m}C_{bk}W_{bk}$$

where *b* = Shift type variable.

*b* represents the shift type, integrating both cost and workforce considerations into the optimisation process [31]. Workforce scheduling models inherently grapple with the dual objectives of cost minimization and productivity maximisation. The inclusion of the variable $C_{bk}$ in optimisation problems underlines the adaptability of IP in tailoring solutions to specific operational goals. This variable’s strategic deployment enables adjustments to scheduling models, reflecting the dynamic interplay between cost efficiency and service quality.

The specific focus of this research on shift balance leverages the previous week’s data to inform the scheduling model and stresses the importance of historical patterns in optimizing future workforce allocations. This approach aims to achieve an equitable distribution of morning, afternoon, and night shifts, offering a balanced and sustainable work environment [2].

The exploration of these mathematical models reveals the depth and versatility of IP in solving complex workforce scheduling problems. By incorporating historical data and sophisticated scheduling constraints, these models offer structured pathways towards optimising workforce allocations, proving it as an invaluable tool.

## 3. Case Study and Problem Definition

### 3.1. Overview of the Clinic

A specialized Oncology clinic in Greece that provides top-tier care, while representing a critical node in the healthcare landscape. Dedicated to delivering advanced treatments and ensuring optimal patient care through effective staff management and operational practices.

### 3.2. Description of the NRP

At the heart of the clinic’s operational efficiency lies the complex task of NR. The goal is to systematically schedule a diverse and specialised nursing team to maintain continuous, round-the-clock patient care. This section delineates the composition of the nursing staff and the specific constraints that govern their scheduling, reflecting the broader challenges of healthcare workforce management.

#### 3.2.1. Staff Composition and Detailed Scheduling Constraints

The clinic has a team of 13 nurses. Aliases were given to preserve anonymity, as shown in Table 1.

Both Su and De are pivotal in ensuring consistent leadership and oversight, working fixed weekday shifts from 07.00 to 15.00. A critical rule is that they cannot be scheduled off on the same day to guarantee managerial continuity.

The roster includes eight Technological Education (TE) nurses and three Secondary Education (SE) nurses. TE nurses and SE nurses are indispensable for providing comprehensive care. A vital scheduling requirement is that SE nurses must always work alongside a TE nurse to ensure a blend of skills and expertise across all shifts, with the rest of the requirements shown in Table 2.

The clinic adheres to a monthly scheduling system aimed at distributing the three eight-hour shifts (07:00–15:00, 15:00–23:00, 23:00–07:00) among the staff in a balanced manner. According to operational regulations, nurses are entitled to a minimum of 11 h of rest between shifts, although in practice, a 16 h rest period is enforced to enhance recovery and well-being. This scheduling paradigm underscores the necessity for a strategic approach to rostering that harmonises regulatory compliance, individual preferences, and clinical demands (Table 3).

#### 3.2.2. Problem and Rostering Constraints

The aim of the problem is to establish a fair distribution of morning, afternoon, and night shifts, while avoiding back-to-back shifts and ensuring an equal number of working days among nurses throughout the week (for instance, preventing one nurse from working six shifts while another works only two). It is often noted that nurses are scheduled for successive shifts without a minimum 16 h break between them. Moreover, there are situations where only one nurse is assigned to a shift that requires two due to poor scheduling, and conversely, there are times when four nurses are assigned to a shift that could be handled by fewer staff. During December, the schedule was devised initially with the restrictions applied as shown in Table 4, Table 5 and Table 6.

Initially, it is evident from the description that the two head nurses are not part of the primary problem since their hours are fixed (i.e., during breakfast) and thus do not need to be considered as parameters in the model. Consequently, the problem focuses on managing 11 nurses ($N_{0}$–$N_{10S}$). The model’s objective is to solve the scheduling problem on a weekly basis, leveraging data from the preceding week. As the problem is aimed at minimization without any cost variables (such as labour costs), we incorporate, as additional constraints, the number of shifts worked by each nurse in the prior week and, as a variable, the type of shift assigned (morning, afternoon, or night).

Additionally, the general on-call duty may be covered by the two head nurses. The number of on-call shifts they handle is predetermined on a monthly basis. The modeling will proceed in two stages. First, a general model will be constructed, incorporating the weekly constraints, followed by a second stage where the specific constraints for each week will be applied. The data structure results in four distinct weekly scheduling problems, each using the same mathematical framework but subjected to varying limitations. To avoid redundancy, the initial focus will be on solving the problem for the first week. This problem is formulated as an MIP model.

### 3.3. Objective Function

The objective is to minimise the function f(x), subject to the following constraints (Equation (3)):

(3) $$\forall j\in J:\;\left(\sum_{i\in I}X_{i,j}=1\right)\;\wedge \;\left(\sum_{i\in I_{T(j)}}X_{i,j}\geq R_{T(j)}\right)$$

The problem for the first week will be solved using the MIP method, considering shift types (morning, afternoon, night) and the number of shifts from the previous week.

## 4. Methodology and Solution Approach

To address the problem, a combination of optimisation software and a programming language was employed. The software selected was IBM’s ILOG CPLEX Optimisation Studio 12.8 (academic license), which is accessible through the library (IloCplex) and the jar file (cplex.jar, included with the installation of ILOG CPLEX Optimisation Studio). The programming language utilised was Java, though the software also supports Python, C#, and other languages. The Integrated Development Environment chosen for this task was Apache NetBeans 8.2 [32], along with Java version 8 [33]. The mathematical method implemented by the software was the branch-and-cut technique.

Initially, 42 shifts of 8 h each needed to be allocated among 11 employees. We defined i→ each employee and j→ each shift, with the first two shifts on the 1st of the month designated as morning shifts (each day comprises 2 morning, 2 afternoon, and 2 night shifts). Additionally, since i,j are indivisible, it follows that i,j∈Z. The distribution of each (s)hift (from s0–s41) is detailed in Table 7.

The aforementioned number may be adjusted if on-call responsibilities necessitate the presence of an additional nurse during the evening hours of the on-call duty. It is essential to note that, for clarity in code comprehension and for the convenience of programmatic resolution, we assume that the numbering of nurses and shifts commences from 0 rather than 1. Likewise, the same logic applies to shifts. Therefore, we get Equation (4).

(4) $$42=\sum_{i=0}^{10}\sum_{j=0}^{41}X_{ij}$$

$$\begin{array}{c} X_{ij}\in \left\{0,1\right\},\;\forall i\in \left\{0,\ldots ,10\right\},\;j\in \left\{0,\ldots ,41\right\}. \end{array}$$

Each nurse may be reassigned following the conclusion of their shift, ensuring a minimum of 11 h (inclusive of a 2 h break) prior to commencing the subsequent shift. Also, each nurse cannot be assigned more than 5 consecutive shifts (Equation (5)). If this condition is not met, the schedule is considered invalid, and she will have to take a day off the following day.

(5) $$\sum_{j=0}^{41}X_{ij}\leq 5$$

Each shift is covered by exactly one nurse (Equation (6)).

(6) $$\sum_{i=0}^{10}X_{ij}=1$$

$$\begin{array}{c} X_{ij}\in \left\{0,1\right\},\;\forall i\in \left\{0,\ldots ,10\right\},\;j\in \left\{0,\ldots ,41\right\}. \end{array}$$

Let the morning shifts {0, 1,…, 37} belong to set Π and the afternoon shifts {2, 3,…, 39} belong to set *A*, while the night shifts {4, 5,…, 41} belong to the set *B*.

The SE nurses ($N_{8S}$, $N_{9S}$, $N_{10S}$) cannot be scheduled in the same shift without TE nurses. Therefore, two TE nurses cannot be on the same shift together. Thus, the following stands:

$$\begin{array}{cc} X_{ij}+X_{i(j+1)}\leq 1, & \;\forall i\in A,\;j\in B,\\ X_{ij}+X_{k(j+1)}\leq 1, & \;\forall i,k\in A\cup \{8\},\;i\neq k,\;j\in B,\\ X_{ij}+X_{k(j-1)}\leq 1, & \;\forall i,k\in A\cup \{8\},\;i\neq k,\;j\in B. \end{array}$$

Furthermore, the nurses with limited time contracts, $N_{7}$ and $N_{10S}$, can cover at max 4 night shifts per month with no more than 1 night shift per week. Thus, we get Equation (7):

(7) $$\begin{array}{c} \sum_{j\in B}X_{ij}\leq 1,\;\forall i\in \left\{7,10\right\}. \end{array}$$

Concerning the remaining nurses, except for $N_{3}$, who has her own schedule, each one can cover a maximum of 2 night shifts per week. Thus, we get (Equation (8)):

(8) $$\sum_{j\in B}X_{ij}\leq 2,\;\forall i\in \left\{0,1,2,4,5,6,8,9,10\right\}$$

Additionally, the maximum number of consecutive night shifts that a nurse can work within a scheduling period is three (Equation (9)):

(9) $$\sum_{j\in A}X_{ij}\leq 3,$$

The model will be a minimisation model, aiming to ensure balance in shift allocation among the staff. This is done using the weight index for each employee for each shift, $C_{i\Pi },C_{iA},C_{iB}$ (morning, afternoon, night). This estimator-index was determined empirically through trials discussed later and from the feedback provided by the hospital workers.

Making an inquiry for every shift of the upcoming scheduling, it will be shown that the morning, being the most desired and with the largest demand, will not allow a higher shift rate for a nurse to cover the morning shift alone. Consequently, $C_{i\Pi }=1$.

Similarly, a parallel evaluation is conducted for the night shifts. Considering that the night shift (23:00–07:00) is the hardest to cover, based on the feedback from the nurses, it should carry the highest weight. Initially, with trial values of $C_{iB}=1.4$, $C_{iB}=1.3$, or $C_{iB}=1.2$ for each night shift, it was observed that if a nurse covered many night shifts the previous week, there is a risk that the weight of the night shifts for the upcoming week would become too large, and thus, one night shift would be equivalent to 2 or 3 morning shifts. Consequently, it was determined that for each night shift, $C_{iB}=1.1$, with the total shift weight from the previous week calculated as (Equation (10)):

(10) $$C_{iB}=1+0.1\left(\sum_{j\in B}X_{ij}\right)$$

Finally, for the afternoon shift, the weight estimator should lie between the weight of the morning shift ($C_{i\Pi }=1$) and the night shift ($C_{iB}=1.1$) 8 h period. As with the night shift, through trials ($C_{iA}=1.04$, $C_{iA}=1.03$, or $C_{iA}=1.02$), an attempt is made to find the appropriate estimator so that, in cases where a nurse covers many afternoon shifts, it is not considered that the weight of 3 or 4 afternoon shifts is numerically equivalent to the weight of 1 night shift. Thus, it was estimated that, for each afternoon shift, $C_{iA}=1.01$, with the total weight of the shifts from the previous week (Equation (11)):

(11) $$C_{iA}=1+0.01\left(\sum_{j\in A}X_{ij}\right)$$

with

$$\min\sum_{i=1}^{11}\sum_{j\in J_{i}}C_{ij}X_{ij}$$

Note, if $N_{2}$ had worked 2 consecutive afternoons and 3 night shifts during the previous week, for the current week, we would get $C_{2A}=1.02$ and $C_{2B}=1.3$.

In accordance with the schedule of the last scheduling period—end of November in Table 8. Note that the ‘+’ symbol indicates pairs of nurses on the same shift.

We get:

$$\begin{array}{cc} C_{i\Pi } & =1,\;\forall i\in \{0,1,2,3,4,5,6,7,8,9,10\},\\ C_{iA} & =\left\{\begin{array}{cc} 1 & \mathrm{if}\;i\in \{0,2,8\},\\ 1.01 & \mathrm{if}\;i\in \{1,4,5,10\},\\ 1.02 & \mathrm{if}\;i\in \{3,6,9\},\\ 1.03 & \mathrm{if}\;i=7, \end{array}\right.\\ C_{iB} & =\left\{\begin{array}{cc} 1.2 & \mathrm{if}\;i\in \{0,1,3,4,10\},\\ 1.1 & \mathrm{if}\;i\in \{2,6,9\},\\ 1 & \mathrm{if}\;i\in \{5,7,8\}. \end{array}\right. \end{array}$$

So, the minimisation model will be (Equation (12)):

(12) $$\min\sum_{i=0}^{10}\sum_{j=0}^{41}C_{ij}X_{ij},$$

where $X_{ij}\in \left\{0,1\right\}$, i∈{0,10}, and j∈{0,41}.

This finalises the generic modelization. In the next section, the weekly model is presented.

### Weekly Modelling

A minimum number of shifts for each nurse to cover weekly is essential. Since the 11 nurses performing 4 shifts per week cover 44 shifts > 42, we consider that the minimum number of shifts will be 3. However, we will consider that, if a nurse is on call, she will have to work one less shift in the clinic as the same will happen in case of leave. We also believe that if last week someone did less than 5 shifts, the next week they will definitely do 5 (except in cases of leave—on-call duty). Therefore, the minimum number of shifts will be introduced as a final restriction. In addition, in cases such as $N_{2}$ or $N_{0}$, who has many days off this week, the variables representing the shifts she will cover this week are not eliminated but reset for the sake of flexibility. The model should be flexible in terms of changes each week and able to reflect nurse-specific considerations, such as leave periods, requested off-days, and previously worked shifts. To that end, we incorporate these factors as follows:

Predefined Assignments: Some nurses have fixed assignments based on external factors (e.g., personal requests or coordination with family schedules). For any nurse, *i*, who must work a specific set of shifts, $J_{i}^{\mathrm{fixed}}$, we directly set (Equation (13)):

(13) $$X_{i,j}=1\;\forall j\in J_{i}^{\mathrm{fixed}}.$$

Unavailable Shifts: If nurse *i* has requested off-days, leave, or cannot be scheduled due to constraints (e.g., adjacency to an on-call night shift), we define $J_{i}^{\mathrm{off}}$ as the set of shifts they cannot cover (Equation (14)):

(14) $$X_{i,j}=0\;\forall j\in J_{i}^{\mathrm{off}}.$$

Minimum Workload Requirements: After accounting for leaves and on-call duties, each nurse, *i*, is assigned a minimum required number of shifts, $r_{i}^{\min}$, for the week (e.g., 3 or 4). We enforce (Equation (15)):

(15) $$\sum_{j=0}^{41}X_{i,j}\geq r_{i}^{\min}.$$

By encapsulating individualised constraints into sets ($J_{i}^{\mathrm{fixed}},J_{i}^{\mathrm{off}}$) and parameters ($r_{i}^{\min}$), we accommodate personal schedules, leaves, rest requirements, and workload balancing within a single, flexible framework. In line with that, Table 9 presents nurse individualised constraints according to available data.

## 5. Results

The application of a Java-based optimisation algorithm to the nurse scheduling problem at the Clinic of Pathological Oncology has significant improvements. To provide a comprehensive evaluation, Table 10 presents the original, pre-optimisation schedule, which served as the baseline, while Table 11 illustrates the new nurses’ schedules, highlighting the optimisation results achieved.

### 5.1. Key Findings

1. Optimisation Outcome: The process culminated in an optimal solution characterised by an objective function value of 43.69. This denotes a significant enhancement in scheduling efficiency, ensuring an equitable distribution of shifts among nursing staff while meeting all operational and individual constraints. Thus, the optimised schedule demonstrated significant measurable improvements, as shown in Table 12.
2. Comprehensive Shift Coverage:
  - Morning shifts are adequately staffed with the Su, the De (which are not part of the problem), and at least two additional nurses, guaranteeing leadership oversight and operational readiness.
  - Afternoon and night shifts are consistently staffed with at least one TE nurse each, alongside another nurse, fulfilling the clinic’s requirement for specialised care around the clock.
3. Adherence to Constraints: The optimisation algorithm meticulously adhered to a complex set of scheduling constraints, including:
  - Prohibiting consecutive shifts for individual nurses to ensure adequate rest.
  - Limiting the number of shifts per nurse to a maximum of five per week to prevent overworking.
  - Integrating individual availability requests and preferences into the scheduling process without compromising operational integrity.
4. Improvement Over Original Schedule: The optimised schedule presents a stark improvement over the original roster by:
  - Eliminating instances of overstaffing and ensuring that each shift is covered by the exact number of required nurses.
  - Addressing previous scheduling inefficiencies, such as the misallocation of TE nurses or failure to meet skill mix requirements for each shift. The SE do not work together at the same 8-h shift and are complemented by the TE.
5. Balanced Workload Distribution: The algorithm ensures a fair and balanced distribution of shifts among all nurses, which:
  - Avoids overburdening any single nurse with an excessive number of night or consecutive shifts.
  - Promotes job satisfaction and well-being by respecting individual work–life balance needs and preferences.
  - The contracted nurses can cover at most one night shift per week, while the others can cover a maximum of two night shifts—except for N3 as in Table 13. Furthermore, it is observed that no employee works two consecutive shifts without at least 16 h between the end of one and the start of the next.
  - It is also understood that no nurse works more than 5 shifts or fewer than 4 shifts in a week—except for those on general on-call duty or leave, such as N0, N2, and N5 Table 13.
6. Clinic Feedback: Positive feedback was received from the clinic’s staff regarding the optimised scheduling solution. The schedule not only satisfies the clinic’s operational and care delivery requirements but also addresses the staff’s work–life balance needs, marking it as an effective and sustainable approach to NR. Positive informal feedback was received from the clinic’s staff regarding the optimised scheduling solution. While satisfaction was not directly measured, the improvements align with established indicators of staff satisfaction. The optimised schedules achieved compliance with regulatory requirements for rest periods and ensured an equitable distribution of shifts among all nurses—operational enhancements likely contributing to improved staff morale [12,34].
7. Key Observations:
  - The solution efficiently covers all nurse shifts, with a total of 42 shifts, ensuring operational continuity without any uncovered periods.
  - It strictly adheres to the clinic’s nurse-to-shift ratio requirements and, as an indirect result, maintains a high standard of patient care.
  - Feedback from the nursing staff indicates a unanimous acceptance of the schedule, highlighting its success in meeting personal preferences and operational demands. To that end, Su’s feedback stated that the solution was correct and was unanimously accepted and implemented by the staff, with zero implications.

### 5.2. Limitations and Threats to Validity

While this study on optimizing nurse rostering shows promising results, several limitations and validity concerns should be flagged.

Data Limitations: The model’s success relies on data regarding nurse availability, patient needs, and workload. Data noise can impact the ability to generate optimal schedules, impacting how well the findings reflect real-world conditions.

Real-time Adaptation: The current model does not yet handle real-time scheduling changes. Its effectiveness could be decreased if it cannot promptly respond to changes in staffing needs or in patient demands.

The Human Factor: The model accounts for work-hour constraints and nurse preferences but may not fully capture unexpected human factors like illness or exhaustion. In that sense, it can be less flexible when responding to last-minute changes and potentially impacting the overall staff satisfaction. To address this, structured surveys or interviews could be incorporated to systematically evaluate nurse perceptions of roster fairness, workload distribution, and overall satisfaction. Such feedback mechanisms would complement the operational metrics, providing a more comprehensive understanding of the model’s impact on workforce well-being and identifying areas for further improvement.

Specific Context: Conducted in a specific oncology setting, the model was tailored to address high patient care urgency through the optimal organization of the nursing staff. However, the results may not be directly communicable to other healthcare departments where the needs might be different. To do so, further adjustments and testing are required.

Scalability Concerns: Although the developed model showcased potential scalability, the complexity could grow substantially when applied to larger healthcare departments. Something like that can lead to diminishing processing times or even reduced performance in real-time applications, thus confining its use in larger or more fast-paced environments/settings.

Although this study offers a foundation for optimizing nurse schedules in oncology settings, addressing these limitations will be necessary to broaden the applicability prospects and enable real-world use. Thus, Section 6 builds upon these and discusses future directions.

## 6. Future Research Directions

New methods propose approaches that can improve NR optimisation further. Starting with generic algorithms, a genetic algorithm has been explored to solve the nurse scheduling problem in crisis situations, such as during COVID-19 [35]. The authors in [36] highlight the effectiveness of hybrid approaches combining, for example, IP and Constraint Programming (CP) to address the highly constrained NRP. In other cases, researchers have proposed hybrid approaches for the NRP, combining MIP with deep neural network-assisted heuristics and recurrent neural networks, outperforming other pieces of research in terms of benchmarks [37,38]. The following sections attempt to categorise similar future trends.

### 6.1. Incorporation of ML Techniques and Others

ML offers promising extensions to traditional NR optimisation, particularly in terms of predictive capabilities and real-time adaptability [39]. For example, using predictive algorithms based on historical data, similar to those in energy forecasting [40,41,42], might enhance the value of OR in healthcare even more. Such data-driven methods can enhance decision-making, leading to better scheduling and improved patient care. Thus, one direction for future work would be to incorporate supervised learning to predict nurse preferences and availability based on historical data, improving the scheduling system’s ability to align nurse shifts with personal preferences. This would enhance both staff satisfaction and operational efficiency by reducing conflicts between organizational requirements and individual nurses’ needs [43].

The authors in [44] address the NRP differently, using a two-stage approach. The first stage combines Monte Carlo Tree Search with Hill Climbing to find feasible solutions by satisfying hard constraints. A novel constant C value is proposed to balance search diversification and intensification in MCTS. The second stage improves the solution using Iterated Local Search with Variable Neighbourhood Descent, introducing unique neighbourhood structures and a perturbation strategy to escape local optima. Computational results on the Shift Scheduling dataset report the best new solutions for several instances. In [45], the researchers suggest that partitioning NRP instances into manageable sub-problems and applying sequential optimisation could be further enhanced with ML techniques to adaptively optimise large, complex NRPs. Finally, another work [46], addresses the NRP using a hyper-heuristic approach that combines Reinforcement Learning (RL) with Simulated Annealing (SA) and Reheating. This method improves scheduling by considering multiple constraints such as labour regulations, hospital policies, and nurse availability. The study, conducted in Norwegian hospitals, demonstrates an 82% improvement in solution quality, outperforming other algorithms such as Simple Random-Hill Climbing, Reinforcement Learning-Hill Climbing, and Reinforcement Learning-Simulated Annealing.

### 6.2. Enhancing Dynamic Capabilities for NR

The enhancement of NR models necessitates additional advancement in dynamic scheduling systems to address the immediate requirements of healthcare settings. The existing frameworks for MIP and ILP, although proficient, exhibit limitations in addressing dynamic constraints, including unexpected nurse absences or varying patient loads. Future research ought to expand these models through the incorporation of stochastic programming, thereby enabling the system to more effectively manage uncertainties by dynamically adjusting schedules in response to real-time feedback regarding nurse availability and patient demand [31]. Another piece of research explored further enhancement of hybrid algorithms, such as combining MIP-based heuristics with metaheuristic methods like SA, to improve solution quality for the NRP [43]. An extended NR model was introduced [47], incorporating unit assignments alongside nurse, day, and shift allocations, addressing the complexities of real-world scenarios where not every nurse can be assigned to every unit due to varying skills and experience. The study also presents a matheuristic solution approach that combines IP for generating initial schedules with Discrete Particle Swarm Optimisation (PSO) for further improvement, ensuring feasibility and near-optimal solutions. In [48], novel strategies for the nurse re-rostering problem are stated, which occurs when unforeseen events disrupt existing schedules and focus on the relaxation of different problem parameters to quickly reconstruct feasible rosters. The integration of accurate optimisation methods can significantly enhance the resilience of scheduling systems, thereby ensuring that optimal solutions maintain stability in the face of variable conditions [43,49].

### 6.3. Real-Time Scheduling, Adaptive Systems, and Public Perception

The development of real-time scheduling systems is critical for managing the complex and unpredictable nature of NR, particularly in oncology departments where patient demand fluctuates rapidly. Future research should focus on implementing adaptive scheduling models that adjust in real time based on nurse availability, patient admissions, and other external factors. By linking these models to real-time data feeds, such as electronic health records, the system could update schedules automatically, reducing the reliance on manual updates by administrators [10].

Combining systematic IP with adaptive algorithms like SA can improve NR in real time. A systematic two-phase approach for NR was developed, which first determines the workload distribution for each nurse and day, followed by the assignment of specific shifts using IP [45]. The approach was applied in the context of the First International Nurse Rostering Competition (INRC2010), where the problem instances were partitioned into sub-problems and solved sequentially. The method’s success was demonstrated by achieving the best results in the competition. In [50], the authors applied SA to a multi-level NRP in hemodialysis services, where nurses with different qualifications must be assigned to various roles, such as in-charge nurse, dispensing nurse, and treatment nurse. The research formulated a 0-1 IP model and used a heuristic algorithm to satisfy both the demand for nurses and their preferences regarding shifts and roles. This adaptive approach successfully addressed the complexity of the problem, ensuring better nurse allocation in a critical healthcare environment.

Another aspect under consideration to enhance NR in oncology departments could be to recognize the impact of social media on public health perceptions and patient engagement [51]. This dynamic can influence staffing decisions, as improved communication with patients through social media may help create schedules that better align with their needs and preferences. Additionally, sentiment analysis from social media discussions on various topics [52] can offer valuable insights into patient concerns, enabling more responsive and effective scheduling strategies for nursing staff.

Future work can also deal with the aspect of cost improvements. The authors in [53] introduce a novel methodology for cyclic preference scheduling using a branch-and-price algorithm to balance individual nurse preferences with cost minimization. The research highlights the growing trend toward cyclic schedules, which are easier to manage, more stable, and generally perceived as equitable in comparison to generating new rosters each period. The proposed approach, which solves instances with up to 200 nurses within minutes, suggests that future research should explore cyclic scheduling models further to enhance stability and manageability in dynamic healthcare environments. Similarly, a cyclic scheduling general IP model which can directly or indirectly support cost minimization is proposed in [54]. More specifically, this work proposes a general IP model for cyclic staff scheduling, which is adaptable to various real-world settings, including a glass plant and a continuous care unit, focusing on the sequence constraints and workload balance through cyclic scheduling.

### 6.4. Broader Application to Various Healthcare Departments and Combined Metrics

While the current focus is on optimising NR in oncology clinics, future research could expand these optimisation models to other healthcare departments on a larger scale and also include other metrics. To that end, insights from prescriptive maintenance [55] could be utilised to automate the identification of suboptimal schedules. Each department within a hospital, such as emergency services, surgical units, and outpatient care, has unique operational requirements and constraints. Developing customisable modules that can adapt the existing MIP model to fit the specific needs of different departments would allow for a more holistic and integrated scheduling solution across healthcare facilities [56]. The authors in [57] address the challenge of constructing nurse duty schedules for large hospitals, balancing staff availability with individual preferences and fairness. Using a tabu search approach, the study developed a decision support system (NuRoDSS) for Stikland Hospital, a large psychiatric facility, demonstrating the scalability of optimisation methods in nurse scheduling. Similarly, another work [58], describes a hybrid approach to nurse scheduling that combines modern heuristic methods with classical IP models, specifically knapsacks, network flow models, and tabu search, to solve a real-world NRP at a major UK hospital.

Combined with the above, metrics concerning patient outcomes or satisfaction scores from both patients and staff could also be included in future research to further strengthen the validity and value of the proposed solutions.

## 7. Conclusions

This study has addressed the complex challenge of optimizing NR in oncology clinics through the application of mathematical optimisation techniques. By employing MIP models, the research demonstrated improvements in operational efficiency, nurse satisfaction, and resource allocation. The results show the importance of using precise mathematical tools to navigate the complexities of healthcare staffing, particularly in settings where patient needs are highly variable.

Building on this, the research investigates the application of ILP and hybrid techniques to adjust to both small- and large-scale rostering challenges. While focused on oncology clinics, the methodology may be extended to broader healthcare contexts, offering versatile solutions for staff allocation in uncertain environments. Dynamic scheduling systems incorporating ML and RL offer the potential for real-time adaptability and data-driven decision-making. Hybrid models, such as those combining ILP with metaheuristic algorithms like Variable Neighbourhood Search (VNS), optimise flexibility and computational efficiency [17,59,60]. Stochastic optimisation further strengthens the ability to respond in a dynamic way to changes in nurse availability and patient needs, paving the way for fair, efficient, and patient-focused staffing systems.

## Figures and Tables

**Table 1:** Clinic nursing team.

Nurse Identifier	Role
Su	Supervisor (Nursing Supervisor)
De	Deputy (Assistant Nursing Supervisor)
$N_{0}$	TE Nurse
$N_{1}$	TE Nurse
$N_{2}$	TE Nurse
$N_{3}$	TE Nurse
$N_{4}$	TE Nurse
$N_{5}$	TE Nurse
$N_{6}$	TE Nurse
$N_{7}$	TE Nurse
$N_{8S}$	SE Nurse
$N_{9S}$	SE Nurse
$N_{10S}$	SE Nurse

**Table 2:** Roles and key requirements.

Role/Group	Staff Members	Critical Scheduling Requirements
Leadership	Su, De	Both cannot be off on the same day. Fixed weekday shifts (07:00–15:00)
TE Nurses	$N_{0}$, $N_{1}$, $N_{2}$, $N_{3}$, $N_{4}$, $N_{5}$, $N_{6}$, $N_{7}$	TE nurses must work alongside SE nurses on all shifts
SE Nurses	$N_{8S}$, $N_{9S}$, $N_{10S}$.	Must always work with at least one TE nurse

**Table 3:** Basic monthly scheduling.

Shift Timing	Rest Period	Notes
07:00–15:00	Minimum 16 h	Balanced distribution of shifts
15:00–23:00	Minimum 16 h	Ensures adequate recovery
23:00–07:00	Minimum 16 h	Aligns with operational regulations

**Table 4:** Nurse shift and duty scheduling overview.

Time Period	Shift Timing	Staffing Requirements
Weekdays		
Morning Hours	07:00–15:00	Su, De, and a minimum of 2 nurses
Afternoon Hours	15:00–23:00	2 nurses, at least one must be TE
Evening Hours	23:00–07:00	2 nurses, at least one must be TE
Public Holidays		
Morning	07:00–15:00	2 nurses, at least one must be TE
Afternoon	15:00–23:00	2 nurses, at least one must be TE
Evening	23:00–07:00	2 nurses, at least one must be TE
Public Holiday Duration	00:00–24:00	Defined from 00:00 to 00:00 the next day
Monthly Shift Coverage	–	62 h each for morning, afternoon, and evening
Night Shift (Extreme Case)	23:00–07:00	1 TE nurse minimum
General On-Call Duty	Various	2 nurses always present
Additional Scheduling Rules		
Weekly Off-Days	–	If a nurse works on a weekend, they don’t work during the week
Max Shifts per Week	–	5 shifts per nurse
Max Afternoon Shifts	–	3 shifts per week
Max Night Shifts	–	2 shifts per week
Public Holidays	–	December 25th, 26th, January 1st
General On-Call Dates	–	1st, 5th, 9th, 13th, 17th, 21st, 25th, 29th December

**Table 5:** Nurse scheduling requests and constraints.

Nurse	Time Period	Requests and Constraints
Su	07:00–15:00 (Weekdays)	Always works breakfast hours, off on weekends. Public Holidays on 25/12 and 26/12. Leave from 11/12 to 15/12
De	07:00–15:00 (Weekdays)	Works breakfast hours, off on weekends. Public Holiday on 25/12 and 26/12
$N_{0}$	23:00–07:00 (4/12)	On-call evening 4/12, can work mornings or take a day off. Parental leave on 7/12. Off on 9/12, 10/12, 23/12, 24/12. Holiday on 25/12 and 26/12
$N_{1}$	07:00–15:00 (10/12, 23/12, 25/12, 26/12)	Off on 5/12, 6/12, 7/12. On-call afternoon 11/12. Breakfast shift on 10/12, 23/12, 25/12, 26/12
$N_{2}$	07:00–15:00 (Various)	Leave from 4/12 to 8/12. Off on 2/12, 3/12, 9/12, 10/12, 30/12, 31/12. On-call afternoon 20/12
$N_{3}$	–	Special schedule adjusted to husband’s program (Fixed by herself)
$N_{4}$	07:00–15:00 (Various)	Off on 1/12. Collected days off from 14/12 to 20/12 and prefers fewer afternoon shifts.
$N_{5}$	07:00–15:00 (Various)	Leave on 1/12 and 4/12. Off on 2/12 and 3/12
$N_{6}$	07:00–15:00 (24/12)	Requested to work in the morning on 24/12
$N_{7}$	23:00–07:00 (4 shifts)	Contract worker. Can work 4 evening shifts per month and 2 holidays. No afternoon shifts on Thursdays or Fridays
$N_{8S}$	07:00–15:00, 23:00–07:00 (25/12)	Off on 23/12, 24/12. Works morning and evening on 25/12, and evening on 26/12. Afternoon shift on 31/12
$N_{9S}$	07:00–15:00 (Mondays, Tuesdays)	Prefers not to work afternoons on Mondays and Tuesdays. Preferably works breakfast hours or takes a day off
$N_{10S}$	23:00–07:00 (4 shifts)	Contract worker. Can work 4 evening shifts per month and 2 shifts on holidays

**Table 6:** Additional nurse scheduling information.

Aspect	Details	Notes
Regular Leave Days	25 days	Permanent staff
Solemn Declaration Absence	4 days per year	Permanent staff
Educational Licenses	–	Not specified
Double Shift Rule	07:00–15:00 and 23:00–07:00	Followed by 23:00–07:00 shift or a day off
Night Shift	22:00–06:00	Defined time for night shifts
Public Holidays	00:00–24:00	Duration from midnight to midnight
Regular Leave Days Counting	–	Requires days off before and after the leave (preferable)
First Day After Leave	07:00–15:00 or Double Shift	Must be a morning or double shift
Days Off Per Week	2 days	Not necessarily within the same week
Holiday Work Compensation	–	Entitled to an extra day off
Double Shift After Afternoon	–	No double shift (07:00–15:00 and 23:00–07:00) after the afternoon (15:00–23:00)

**Table 7:** Shift schedule for the first week of December.

December (1st Week)							
**Day**	**Fri**	**Sat**	**Sun**	**Mon**	**Tue**	**Wed**	**Thu**
Morning	$s_{0}$, $s_{1}$	$s_{6}$, $s_{7}$	$s_{12}$, $s_{13}$	$s_{18}$, $s_{19}$	$s_{24}$, $s_{25}$	$s_{30}$, $s_{31}$	$s_{36}$, $s_{37}$
Afternoon	$s_{2}$, $s_{3}$	$s_{8}$, $s_{9}$	$s_{14}$, $s_{15}$	$s_{20}$, $s_{21}$	$s_{26}$, $s_{27}$	$s_{32}$, $s_{33}$	$s_{38}$, $s_{39}$
Night	$s_{4}$, $s_{5}$	$s_{10}$, $s_{11}$	$s_{16}$, $s_{17}$	$s_{22}$, $s_{23}$	$s_{28}$, $s_{29}$	$s_{34}$, $s_{35}$	$s_{40}$, $s_{41}$

**Table 8:** ID distribution per shift for November’s last week.

Last Week of November							
**Day**	**Fri**	**Sat**	**Sun**	**Mon**	**Tue**	**Wed**	**Thu**
Morning	$N_{2}$ + $N_{8S}$, $N_{3}$ + $N_{7}$	$N_{2}$, $N_{7}$	$N_{0}$, $N_{1}$ + $N_{7}$	$N_{0}$ + $N_{2}$, $N_{4}$ + $N_{6}$	$N_{2}$, $N_{6}$ + $N_{9S}$	$N_{1}$, $N_{6}$ + $N_{10}$	$N_{0}$ + $N_{7}$, $N_{2}$ + $N_{6}$ + $N_{8S}$
Afternoon	$N_{5}$, $N_{9S}$	$N_{6}$, $N_{9S}$	$N_{10S}$, $N_{6}$	$N_{1}$, $N_{7}$	$N_{3}$, $N_{7}$	$N_{3}$, $N_{7}$	$N_{10S}$, $N_{4}$
Night	$N_{1}$, $N_{10S}$	$N_{2}$, $N_{3}$	$N_{0}$, $N_{3}$ + $N_{10S}$	$N_{0}$, $N_{4}$	-, $N_{4}$	$N_{1}$, $N_{9S}$	$N_{8S}$, $N_{6}$

**Table 9:** Nurse Individualised Constraints.

Nurse	Constraint Description
** $N_{0}$ **	She is scheduled as the on-call nurse on December 4 (23:00–07:00), which prevents her from working 16 h before or after that shift. She also requested parental leave on December 7, making her unavailable for the previous night’s shift. As a result, her minimum shifts this week are 3, calculated from 7 days minus 2 off days, 1 on-call day, and 1 leave day.
$N_{1}$	She requested days off on December 5 and 6, and Friday, December 7, totalling three days. Therefore, she cannot be assigned a shift on December 5.
** $N_{2}$ **	She requested a leave of absence from the 4th to the 8th of the month (the 4th to 7th impacting this week) and days off on the 2nd and 3rd. She also cannot be assigned to the previous night’s shift. Therefore, the minimum number of shifts she must work this week is 1, calculated as 7 days minus 2 off days and 4 leave days.
** $N_{3}$ **	Sets her schedule based on her husband. She will work on the 1st and 2nd of the month.
$N_{4}$	She requested to have December 1 off.
$N_{5}$	She requested days off on December 1 and December 4, and additional leave on December 2 and 3. Therefore, she must work 3 shifts this week (7 days–2 off days–2 leave days).
$N_{6}$	She does not have any constraints this week.
$N_{7}$	She has requested not to be scheduled for the afternoon shift (15:00–23:00) on Thursday and Friday.
$N_{8S}$	She has no restrictions this week.
$N_{9S}$	She has requested days off on Monday and Tuesday, meaning she cannot work the night shift on Sunday.
$N_{10S}$	She worked the 15:00–23:00 shift on Thursday of the previous week (November 3), so she cannot work the morning shift on Friday.
All Nurses except for $N_{0}$, $N_{2}$ and $N_{5}$	Must work at least 4 shifts during the week.

**Table 10:** Shift schedule for the first week of December with nurse IDs before the solution.

December (First Week)							
**Day**	**Fri**	**Sat**	**Sun**	**Mon**	**Tue**	**Wed**	**Thu**
Morning	$N_{1}$, $N_{2}$.$N_{7}$	$N_{9S}$, null	$N_{4}$, $N_{8}$	$N_{0}$.$N_{10S}$, $N_{9S}$	$N_{5}$, $N_{6}$.$N_{7}$	$N_{3}$.$N_{8S}$, $N_{5}$.$N_{6}$	$N_{4}$, $N_{8S}$
Afternoon	$N_{0}$, $N_{9S}$	$N_{4}$, $N_{10S}$	$N_{6}$, $N_{9S}$	$N_{3}$, $N_{8S}$	$N_{3}$, $N_{8S}$	$N_{4}$, $N_{9S}$	$N_{9S}$, $N_{5}$
Night	$N_{1}$, $N_{3}$.$N_{8S}$	$N_{1}$, $N_{3}$	$N_{4}$, null	$N_{0}$.$N_{10S}$, $N_{4}$	GE, $N_{7}$	$N_{7}$, $N_{6}$	$N_{3}$, null

**Table 11:** Shift schedule for the first week of December with nurse IDs after the solution.

December (First Week)							
**Day**	**Fri**	**Sat**	**Sun**	**Mon**	**Tue**	**Wed**	**Thu**
Morning	$N_{1}$, $N_{9S}$	$N_{1}$, $N_{7}$	$N_{9S}$, $N_{7}$	$N_{10S}$, $N_{7}$	$N_{10S}$, $N_{5}$	$N_{6}$, $N_{10S}$	$N_{6}$, $N_{1}$
Afternoon	$N_{2}$, $N_{0}$	$N_{4}$, $N_{0}$	$N_{1}$, $N_{0}$	$N_{1}$, $N_{3}$	$N_{8S}$, $N_{3}$	$N_{4}$, $N_{8S}$	$N_{4}$, $N_{8S}$
Night	$N_{8S}$, $N_{3}$	$N_{6}$, $N_{3}$	$N_{8S}$, $N_{4}$	$N_{6}$, $N_{4}$	$N_{7}$, $N_{9S}$	$N_{5}$, $N_{9S}$	$N_{5}$, $N_{3}$

**Table 12:** Quantitative evaluation of optimisation results.

Quantitative Metrics Before and After Optimisation						
**Metric**	**Rest Violations**	**Skill Mix Compliance**	**Workload Balance**	**Overstaffing**	**Understaffing**	**Objective Function Value**
Before Optimisation	3 violations	85% of shifts	2.3 shifts	2 instances	3 instances	239.5
After Optimisation	0 violations	100% of shifts	0.9 shifts	0 instances	0 instances	43.69

**Table 13:** Number of shifts per Nurse.

Nurse	Number of Shifts
$N_{0}$	3
$N_{1}$	4
$N_{2}$	1
$N_{3}$	5
$N_{4}$	5
$N_{5}$	3
$N_{6}$	4
$N_{7}$	4
$N_{8S}$	5
$N_{9S}$	4
$N_{10S}$	4
Total shifts	42

## Data Availability

The data supporting the reported results are available upon reasonable request from the corresponding author. The data are not publicly available to preserve anonymity.

## Abbreviations

The following abbreviations were used in this manuscript:

CP	Constraint Programming
CPLEX	IBM’s ILOG CPLEX Optimisation Studio
De	Deputy
ILP	Integer Linear Programming
IP	Integer Programming
LP	Linear Programming
MCTS	Monte Carlo Tree Search
MIP	Mixed-Integer Programming
MILP	Mixed-Integer Linear Programming
ML	Machine Learning
NRP	Nurse Rostering Problem
NR	Nurse Rostering
OR	Operations Research
PIP	Pure Integer Programming
RL	Reinforcement Learning
SA	Simulated Annealing
SE	Secondary Education (Nurse)
Su	Supervisor
TE	Technological Education (Nurse)
VNS	Variable Neighbourhood Search

## Nomenclature

The following nomenclature was used in this manuscript:

$X_{ij}$	Binary variable indicating if nurse *i* is assigned to shift *j*
*i*	Nurse index
*j*	Shift index
*I*	Set of all nurses
*J*	Set of all shifts
Π	Set of morning shifts
*A*	Set of afternoon shifts
*B*	Set of night shifts
$C_{i\Pi }$	Weight for nurse *i* assigned to morning shifts
$C_{iA}$	Weight for nurse *i* assigned to afternoon shifts
$C_{iB}$	Weight for nurse *i* assigned to night shifts
$I_{T(j)}\subseteq I$	Subset of nurses qualified for shift type T(j)
$R_{T(j)}$	Required minimum number of qualified nurses for shift type T(j)
T(j)	Type of shift *j* (morning, afternoon, or night)
$\sum X_{ij}$	Total shifts assigned to nurse *i*
$\sum X_{i\Pi }$	Total morning shifts assigned to nurse *i*
$\sum X_{iA}$	Total afternoon shifts assigned to nurse *i*
$\sum X_{iB}$	Total night shifts assigned to nurse *i*
Su	Supervisor nurse
De	Deputy nurse
TE	Technological Education nurse
SE	Secondary Education nurse
$\min\sum C_{ij}X_{ij}$	Objective function to minimize weighted shifts across all nurses
